# Focused extracorporeal shockwave therapy for the treatment of low back pain: a systematic review

**DOI:** 10.3389/fmed.2024.1435504

**Published:** 2024-08-29

**Authors:** Dilyan Ferdinandov

**Affiliations:** ^1^Department of Neurosurgery, Faculty of Medicine, Medical University - Sofia, Sofia, Bulgaria; ^2^Clinic of Neurosurgery, St. Ivan Rilski University Hospital, Sofia, Bulgaria; ^3^Vertebra Medical Center, Sofia, Bulgaria

**Keywords:** low back pain, treatment, focused shockwave therapy, randomized controlled trial, systematic review

## Abstract

**Introduction:**

Low back pain (LBP) is a common condition affecting up to 84% of people in their lifetime, with a prevalence of 11.9% and a high recurrence rate within the first year. Furthermore, chronic low back pain syndrome has been described in up to 7%, making it a significant health and socioeconomic problem. Among nonoperative treatment options, the recently used focused extracorporeal shockwave therapy (ESWT) devices generate waves that converge at a precise depth in the body, thereby revealing the potential to affect pathology remotely from the contact surface. The article aims to present a systematic literature review with a critical discussion on treating low back pain using this modality.

**Methods:**

A search for randomized controlled trials (RCT) of focused ESWT for low back pain published before April 1, 2024, in PubMed, Web of Science, Scopus, Google Scholar, and trial registries (WHO International Clinical Trials Registry Platform and ClinicaTrials.gov) was performed.

**Results:**

Only three studies against conservative treatment comprising 94 patients met the selection criteria and were further analyzed. Comparative clinical studies regarding the effectiveness of radial and focused ESWT for low back pain were missing. The results revealed that all treated patients had significantly reduced pain and improved functional impairment immediately after the procedures and 1 month later. At the third month time point, the pain levels remained better in the experimental than in the control group without achieving statistical significance. None of the studies had a long-term follow-up.

**Conclusion:**

Focused ESWT is a modern physiotherapeutic method that can potentially treat a broad spectrum of conditions responsible for low back pain. Despite the small number of low-evidence studies, there is sufficient data on the effectiveness and safety of this therapeutic modality. With future well-designed trials, the bias risks would be diminished, the indications for its use would expand, and the treatment protocols would be clarified.

## Introduction

Low back pain (LBP) is a common condition that affects up to 84% of people in their lifetime and has a prevalence of 11.9% (1). In most cases, the acute episode will resolve in 6 weeks, but between 25 and 78% of patients will have recurrence within the first year (2–4). Chronic low back pain syndrome has been described in up to 7% and is defined as symptoms lasting more than 12 weeks, making it a significant health and socioeconomic problem (5).

LBP treatment requires an interdisciplinary approach that includes modalities ranging from bed rest, manual and kinesiotherapy, pharmacological treatment, physical methods, and a broad spectrum of minimally invasive interventions before open surgery (6, 7). However, only 31–47% of patients with chronic LBP will have relief within 1 year, which raises the need for new approaches (8).

Among nonoperative treatment modalities, extracorporeal shock wave therapy (ESWT) is a noninvasive procedure using acoustic waves generated outside the body and targeted in depth on the pathology. This type of energy has a described biological effect at the cellular, tissue, and organ levels. Still, the exact mechanisms of impact on the structures of the musculoskeletal system and the adjacent neural elements remain unclear. Low energy levels have mechanical stimuli and positive effects, leading to cell migration, proliferation, and differentiation. Reduced swelling and infiltration of inflammatory cells in the tissues were also found (9). High energy levels are believed to have shear stress and are destructive (10). Pain relief is thought to result from hyperstimulation of nerve endings (11). In addition to the above, given the importance of paravertebral muscle spasm in degenerative spine pathologies, ESWT has been found to reduce spasticity, decrease connective tissue stiffness, and stimulate nitric oxide synthesis, leading to improvement in neuromuscular transmission and vasodilation (12).

From a therapeutic point of view, radial and focused extracorporeal shock wave therapy (ESWT) is considered. The radial one produces pressure waves that diverge deep into the tissues, with low velocity and peak pressure, depleting away from the applicator (9). Thus, the effects are primarily superficial. The FDA approved the use of radial ESWT devices for the treatment of plantar fasciitis in 2000 and lateral epicondylitis in 2003 (13). The indications, therapeutic protocols, and results regarding musculoskeletal disorders are clear to date. In contrast, the newer focused ESWT generates waves that converge at a precise depth in the body, thereby revealing the potential to affect pathology that is remote from the contact surface (10). The main power generators used are piezoelectric, electromagnetic, and electrohydraulic (13). The physical effects of focused ESWT are related to the energy delivered to a specific cross-section, defined as energy flux density (EFD, mJ/mm^2^).

To date, many clinical studies have compared the effectiveness of the two types of ESWT for diverse indications. The results show the effectiveness of both therapies despite the different mechanisms on the tissues (14–17) Few studies have addressed the treatment of low back pain using focused ESWT. This work aims to present a systematic literature review with a critical discussion.

## Materials and methods

**Figure 1:**
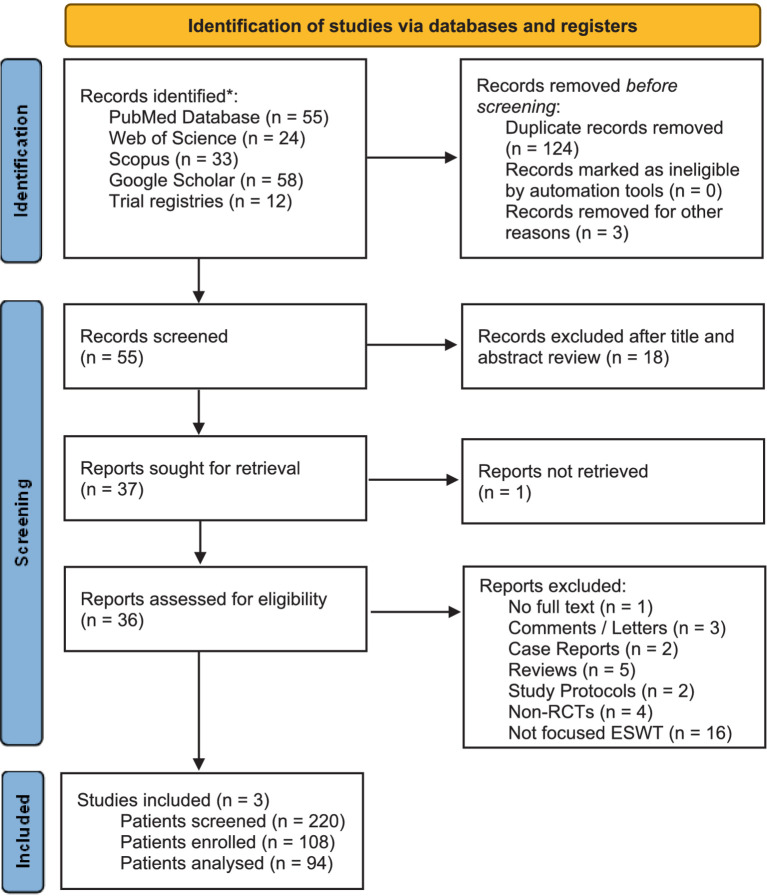
PRISMA diagram presenting the search for randomized controlled trials (RCT) of focused ESWT for low back pain published before April 1, 2024. *No restrictions regarding the year of publication, country of origin, or language were applied.

A search for randomized controlled trials (RCT) of focused ESWT for low back pain published before April 1, 2024, in PubMed, Web of Science, Scopus, Google Scholar, and trial registries (WHO International Clinical Trials Registry Platform and ClinicaTrials.gov) was performed. The following keywords and phrases were used: focused extracorporeal shockwave therapy, ESWT, low back pain, lumbosacral pain, lumbar spine, sacroiliac joint, and facet joint syndrome. Relevant references from the identified articles were further retrieved and analyzed. The PRISMA guidelines were used in preparing this systematic review, and a corresponding diagram is presented here (Figure 1). No restrictions regarding the year of publication, country of origin, or language were applied.

## Results

Following the search strategy, 55 articles were initially identified. By refining the results, 19 clinical studies were extracted. Table 1 presents a list of randomized controlled trials for the treatment of low back pain (LBP) with extracorporeal shockwave therapy (ESWT), which were excluded from further analysis after a detailed review. All these studies report results with radial shockwave or vibrotherapy devices. Only 3 met the criteria for a randomized controlled trial of focused extracorporeal shockwave to treat low back pain. Comparative clinical studies regarding the effectiveness of radial against focused ESWT for low back pain are missing. Table 2 summarizes the basic demographic characteristics, symptoms’ duration, clinical outcome assessment tools, and follow-up periods. Table 3 presents the treatment protocols of the selected studies. Tables 4, 5 summarize the results of the analyzed randomized clinical studies.

**Table 1:** List of randomized controlled trials for the treatment of low back pain (LBP) with extracorporeal shockwave therapy (ESWT), which were excluded from further analysis after a detailed review.

Author	Year	Study	Reason for exclusion
Zheng et al. (26)	2013	ESWT vs. Thermomagnetic therapy in chronic LBP	Radial ESWT device—ShockMaster 500, Gymna, Belgium
Lee et al. (27)	2014	ESWT vs. Conservative physical therapy in chronic LBP	Radial ESWT device—JEST-2000, Joeun Medical, Korea
Han et al. (28)	2015	ESWT vs. Conservative physical therapy in chronic LBP	Radial ESWT device—VITERA, Comed, Korea
Hong et al. (29)	2017	EWST vs. Trigger point injection for the treatment of the quadratus lumborum myofascial pain syndrome	Dornier AR2 with smart focus technology (MedTech, Munchen, Germany)
Nahas et al. (30)	2018	ESWT and exercises vs. Exercises in postpartum LBP	Radial ESWT device—Unknown model, Storz Medical, Switzerland
Schneider et al. (24)	2018	ESWT and myofascial trigger therapy vs. myofascial trigger therapy in chronic LBP	Vibrotherapy—Cellconnect Impulse
Walewicz et al. (31)	2019	ESWT and stabilization training vs. Sham ESWT and stabilization training in chronic LBP	Radial ESWT device—Pro-Shock Waves Pneumatic, Cosmogamma, Indonesia
Çelik et al. (32)	2020	ESWT vs. Sham ESWT in chronic LBP	Electrohydraulic lithotripter—EMD, E1000, C-ARMOR, Turkey
Eftekharsadat et al. (23)	2020	ESWT and stretching exercises vs. Corticosteroid injections and stretching exercises in LBP	Radial ESWT device—enPulsPro, Zimmer MedizinSysteme, Germany
Notarnicola et al. (33)	2020	ESWT vs. Exercises in sacroiliac joint pain	Lithotripter—Minilith SL1, Storz Medical, Switzerland
Guo et al. (34)	2021	ESWT vs. ESWТ and medication therapy vs. Medication therapy in chronic LBP	Radial ESWT device—Swiss DolorClast® EVO BLUE, Switzerland
Lange et al. (35)	2021	ESWT vs. Sham ESWT and medication therapy in acute LBP	Radial ESWT device—Swiss DolorClast® EVO BLUE, Switzerland
Elgendy et al. (36)	2022	ESWT and standard exercise program vs. standard exercise program in chronic LBP	Radial ESWT device—HC Shock Wave, Elettronica Paganis, Italy
Kong et al. (37)	2022	ESWT vs. Laser therapy in chronic LBP	Radial ESWT device—HK.ESWO-AJ, Shenzhen Huikang Medical Apparatus, China
Sun et al. (38)	2022	ESWT comparing different treatment protocols in chronic LBP	Radial ESWT device—enPuls, Zimmer MedizinSysteme, Germany
Wu et al. (39)	2023	ESWT vs. Thermomagnetic therapy in LBP	Radial ESWT device—BHSW Ballistic, Weihai Bohua Medical Equipment Co., China

All these studies report results with radial shockwave or vibrotherapy devices.

**Table 2:** Summary of the randomized controlled trials regarding the basic demographic characteristics, duration of symptoms, assessment tools for the clinical outcome, and follow-up periods.

Author	Year	Study design	Group	Subjects enrolled	Subjects analyzed	Mean age, years	BMI	Symptoms duration, months	Assessment tools	FUP, months
Moon (18)	2017	Prospective, randomized, controller, single-center	ESWT	15	14	54.42 ± 19.05	NS	20.42 ± 11.81	VAS, ODI	1 and 4 weeks
			Sham	15	11	59.18 ± 15.30		17.70 ± 6.81		
Taheri (20)	2021	Prospective, randomized, controlled, single-center	ESWT	19	17	42.5 ± 10.1	27.1 ± 5.5	4.6 ± 1.2	VAS, ODI	1 and 3 months
			Sham	19	15	37.1 ± 11.8	26.8 ± 2.1	5.0 ± 1.2		
Rajfur (21)	2022	Prospective, randomized, controlled, single-blinded, single-center	ESWT	20	19	42.3 ± 13.1	24.3 ± 3.9	57.5 ± 50.9	VAS, LPS, ODI	After the end, 1 and 3 months
			Sham	20	18	45.4 ± 14.0	26.5 ± 3.0	61.8 ± 53.1		

ESWT, focused extracorporeal shockwave group (experimental arm); Sham, sham-intervened group (control arm); VAS, Visual Analogue Scale; ODI, Oswestry Disability Index; LPS, Laitinen Pain Scale; FUP, follow-up period.

**Table 3:** Treatment protocols of the randomized controlled trials.

Author	Year	ESWT	Control	Treatment regimen	Additional treatment	Device
Moon (18)	2017	2000 shocks, 3 Hz frequency, 0.09–0.25 mJ/mm^2^^*^	Sham procedure (0.03 mJ/mm^2^ with parallel probe orientation)	Single session	Refrain from anti-inflammatory medication and other physical modalities	Aries, Dornier MedTech, Germany
Taheri (20)	2021	1,500 shocks, 4 Hz frequency, 0.15 mJ/mm^2^^**^	Sham procedure (sound without energy)	Once weekly for 4 weeks (4 sessions)	Exercise program with muscle stretching and strengthening; oral medications (meloxicam 15 mg/d for 2 weeks; tizanidine 2 mg/d for 10 days)	Aries2, Dornier MedTech, Germany
Rajfur (21)	2022	1,000 shocks, 4 Hz frequency, 0.15 mJ/mm^2^^**^	Sham procedure (absorbing insert)	Twice weekly for 5 weeks (10 sessions)	Stabilization training (45 min, once a day, 5 days a week) with myofascial relaxation, dynamic postural exercises	Duolith SD1, Storz Medical, Switzerland

The additional treatments are described in detail. ESWT, focused extracorporeal shockwave group (experimental arm); Control, sham-intervened group (control arm).

* Energy flux density (EFD) was set to the maximum tolerated by the patient.

** EFD was fixed.

**Table 4:** Baseline characteristics and clinical results for pain (VAS).

Author	Year		Baseline	After treatment	Month 1	Month 3
Moon (18)	2017	ESWT	6.42 (5.19–7.66)	not given	3.64 (2.29–4.99)^*^^,^^#^	
		Sham	Not given	Not given	6.18 (5.34–7.02)^#^	
Taheri (20)	2021	ESWT	6.6 ± 1.8		3.0 ± 2.3^*^	1.8 ± 2.8^**^
		Sham	6.8 ± 1.9		4.6 ± 1.8^*^	1.1 ± 1.5^**^
Rajfur (21)	2022	ESWT	7.2 ± 1.9	1.5 ± 0.6^*^^,^^#^	1.7 ± 1.1^*^^,^^#^	2.0 ± 1.2^*^
		Sham	7.3 ± 1.7	2.9 ± 1.3^*^^,^^#^	3.1 ± 1.7^*^^,^^#^	3.3 ± 1.9^*^

Data is expressed as mean ± SD except for the study of Moon et al., where the 95% confidence interval is presented in brackets.

* Statistically significant difference within groups at the corresponding follow-up time points compared to baseline.

** Statistically significant difference within groups at month 3 compared to month 1.

# Statistically significant difference between groups at each time point.

**Table 5:** Baseline characteristics and clinical results regarding the quality of life because of pain (ODI).

Author	Year	Group	Baseline	After treatment	Month 1	Month 3
Moon (18)	2017	ESWT	17.80 (13.08–22.63)	12.92 (9.19–16.67)	11.28 (7.30–15.28)	
		Sham	Not given	Not given	Not given	
Taheri (20)	2021	ESWT	41.1 ± 21.2		11.9 ± 6.6^*,^^#^	7.1 ± 5.7^**^
		Sham	40.5 ± 19.1		22.9 ± 9.4^*,^^#^	8.9 ± 5.7^**^
Rajfur (21)	2022	ESWT	33.4 ± 6.3	18.3 ± 7.5^*^	17.3 ± 7.1^*^	18.3 ± 6.8^*^
		Sham	32.5 ± 8.6	19.5 ± 6.5^*^	18.7 ± 6.6^*^	19.9 ± 7.4^*^

Data is expressed as mean ± SD except for the study of Moon et al., where the 95% confidence interval is presented in brackets.

* Statistically significant difference within groups at the corresponding follow-up time points compared to baseline.

** Statistically significant difference within groups at month 3 compared to month 1.

# Statistically significant difference between groups at each time point.

Moon et al. (18) published a prospective randomized, sham-controlled, single-center trial on 25 patients with sacroiliac joint pain. The inclusion criteria are clearly defined with symptoms duration of more than 6 months, at least 19 years of age, pain >4 on a 10-cm numeric rating scale localized in the SIJ region, and at least three of five provocation SIJ tests from Patrick’s sign, Gaenslen test, compression test, thigh trust test, and distraction test (19). Among the author’s exclusion criteria were: ESWT administered to any other body lesion; a positive straight leg-raising test; radiologically confirmed lumbar or hip joint pathology; pregnancy; acute pelvic inflammation; and previous SIJ intervention (i.e., corticosteroid injection within the previous 12 months). Participants were instructed to refrain from any other conservative treatments, including medications for pain or physical therapy. Randomization was in blocks of six by a blinded physician using a computerized random number generator. The study protocol included a focused ESWT in a single treatment session comprising 2000 shocks at 3 Hz, though perpendicular to the area probe and energy level 0.09–0.25 mJ/mm^2^. The control group received a single session of sham intervention with a parallel-oriented probe and a noise at every sock, which was delivered with a minimal energy of 0.03 mJ/mm^2^. All patients were blindfolded. A 10-cm VAS type and the ODI were used for evaluations before and 1 and 4 weeks after treatment by a physician blinded to the other procedures. The authors found a significant improvement in the pain score in the fESWT group at week 4 post-treatment compared to the baseline, which was not observed in the control group. Although there was a trend toward improvement from baseline in the ODI regarding the intervened patients, statistical significance was not reached for both groups. Side effects of fESWT were not evident.

Taheri et al. (20) presented the results from a randomized controlled trial on 32 patients with chronic low back pain with a duration of more than 3 months who had never undergone surgery or any other treatment for the last month associated with their disease. Pregnant women and patients with mental or cognitive problems were not included. Among the exclusion criteria were cancer, fractures, infections, disc degeneration resulting from aging or trauma, an unstable medical condition, or uncontrolled systematic diseases. Thirty-eight patients were enrolled and randomly allocated equally to the focused ESWT or control group, nine were not eligible, and three refused. Six subjects were lost during the follow-up due to unwillingness to continue, and 32 study completers were analyzed—17 and 15 from the abovementioned groups, respectively. The protocol included focused ESWT or sham procedure, as well as oral medications and an exercise program for all. The pressure pulses were targeted on the surface trigger points through a contact lubricant, and 1,500 of them were delivered at 0.15 mJ/mm^2^ energy density and 4 Hz frequency. The sessions were once weekly for 4 weeks. Patients in the control group had sham procedures with the same treatment regimen, which had the same sound but without energy applied. All subjects received oral medications (meloxicam 15 mg/daily for 2 weeks and tizanidine 2 mg/daily for 10 days) and fulfilled an exercise program. ODI questionnaire was used to evaluate the degree of functional disability, and the visual analog scale was used to assess the pain at baseline and after 1 and 3 months. Appropriate statistical analysis was performed. The groups were comparable in terms of sex, age, body mass index, duration, and severity of complaints. The pain score decreased during the study period in both groups without statistically significant differences between them. ODI is observed to be the same but with a significantly lower score at 1 month in favor of the interventional arm and not at 3 months.

Rajfur et al. (21) conducted a prospective randomized, single-blind study with a 3-month follow-up regarding the efficacy of focused ESWT in patients with chronic low back pain. Subjects were assigned to real or sham treatments using a computer random number generator. Both groups performed basic exercises to stabilize the spine. The same therapist performed all tests and surveys, and the same physiotherapist performed all treatments and exercises. Patients with MRI-confirmed L5-S1 discopathy (Modic type 3 changes), chronic pain lasting at least 12 weeks, and no spinal surgical interventions were enrolled. Among the exclusion criteria were discopathy beyond the L5-S1 level (Modic type 1 and 2), reduced segmental mobility, other spinal conditions, neurologic deficit, blood coagulation disorders, metal implants at the treatment site, sensory disturbances, mental disorders, cancer, local skin lesions, and infections. The study involved 40 subjects equally allocated in the two homogenous and comparable groups. Three patients were excluded from the statistical analysis—one was lost in the follow-up period from the treatment group and two from the sham procedure group because of taking painkillers. According to the authors, each procedure was performed using the contact method at the lower back, where the most severe pain is localized.

The energy flux density was 0.15 mJ/mm^2^ in 1000 pulses with a frequency of 4 Hz. Treatments were performed twice a week for 5 weeks under ultrasound guidance. Patients from the control group received a sham procedure using a polyethylene-absorbing insert on the top of the applicator with the same audible signals and technical parameters. Identical stabilization training with myofascial relaxation and dynamic postural exercises were performed in both groups 5 days a week. The assessment was done using a visual analog scale (VAS), Laitinen Pain Scale (LPS), and Oswestry Disability Index (ODI) before and after treatment and during follow-up at 1 and 3 months. Appropriate statistical analysis was performed. The groups were comparable in terms of demographic and clinical characteristics. The authors found a significantly greater improvement for the focused ESWT compared to the sham group immediately after treatment and 1 month later but not in the 3-month follow-up in VAS and LPS. This was not evident regarding the ODI scores. Still, the patients in the experimental group had greater improvement.

## Discussion

Considering the available clinical studies, several problems in future designs should be addressed. First of all, the differences in the inclusion and exclusion criteria for subjects in the known series are significant. Many of them are controversial and prone to selection bias. At the same time, if we strictly adhere to them, major patient populations are not covered. Second, uniform treatment parameters have not been established to date. The applied therapeutic protocols are not based on theoretical statements, experimental findings, and practical experience. Lastly, there is a need for objective assessment and reproducible tools regarding the clinical outcome. Thus, even the few low-quality studies are not comparable.

Notably, in the study of Moon et al. (18), 98 patients were assessed for eligibility, of which 39 did not meet the inclusion criteria, and 27 declined participation. From the allocated 30 subjects, 15 in the focused ESWT and 15 in the sham-intervened group, there was one loss for follow-up from each one. Another three patients from the controls were drop-outs due to pain medication intake. Thus, only 25 patients, 14 from the experimental and 11 from the sham-stimulation groups, achieved analysis. The abovementioned poses a significant risk of selection bias. Several points of this study also remain disputable. For example, focused ESWT in another body part is irrelevant to the local procedure in the current area of interest, and such patients might not be excluded. Furthermore, cases with facet joint syndrome encompass a large proportion of the low back pain population. This is an important group, where it is sometimes difficult to differentiate from the pain of sacroiliac joint origin, even with negative imaging findings, and it contributes further to the selection bias.

The study of Taheri et al. (20) has several limitations, including the small number of subjects, as noted by the authors. Out of 50 patients, 12 were excluded, and another six were lost during the follow-up, which implies observational bias. Disc degeneration is stated to be an exclusion criterion, but this is the anatomical substrate of low back pain in most cases. Thus, this point is disputable and unclear. In addition, it is difficult to differentiate the effect of the focused EWST because of the routinely administered drug therapy in all patients.

The randomized controlled trial of Rajfur et al. (21) also has several drawbacks and limitations that have not been discussed by the authors. Some exclusion criteria remain disputable, like implanted cardiac pacemakers. For the study examiners, it is difficult to control the intake of painkillers and anti-inflammatory drugs in patients with pain syndrome. Reduced mobility in the lumbosacral segment is nonsense as an exclusion criterion. In the same context, a discopathy beyond the L5-S1 Modic type 1 and 2 changes remains unclear. Furthermore, ovulation in healthy women included in this study is expected to occur every 4 weeks, which confronts the protocol, and this population of patients should not be included.

Evaluation of the treatment effect in patients with pain syndrome is difficult and, in many cases, subjective. To address this problem, Elgendy et al. (22) published a randomized controlled trial of radial ESWT in chronic low back patients. Therefore, this study is not part of the current analysis. However, the authors evaluated the electromyographic (EMG) activity of trunk muscles (lumbar multifidus and lumbar erector spinae) in the form of root mean square. After electrode placement, the protocol included the application of an appropriate resistance at the scapular region to maintain the maximum isometric muscular contraction three times. Then, the patient was asked to gradually increase the force to reach an absolute maximum and to hold it for 8–10 s. Three maximal isometric extension efforts were performed. Approximately 30 s of rest were given between contractions. EMG sampling frequency in their protocol was 1,000 HZ, and the sensitivity was 500 μs. The total root mean square of the recorded signals was obtained. The authors found that their increase correlates with lower VAS scores for pain. This approach needs to be replicated in further studies.

In a single-blind randomized clinical trial, Eftekharsadat et al. (23) investigated the effect of radial ESWT on patients with low back pain, which is also not included in this analysis. However, the authors present a pressure-pain threshold assessment using a commercially available digital algometer for the myofascial trigger points on quadratus lumborum muscles. Larger values indicate higher pain thresholds. The device has a 1.0 cm^2^ circular flat tip, which was slowly pushed upright to the skin over the trigger points. The exerted pressure was increased gradually until the pain was perceived. The measurements were implemented thrice with 40 s intervals, and the mean value was considered.

Addressing the primary end-point, which is the pain intensity, all future studies for LBP treatment should rely not only on the widely accepted visual analog type of scales. A more detailed assessment could be achieved with the Oswestry Disability Index and the Short Form 36 health survey for quality of life. However, both require active patient participation and, in some cases, the need for assistance from a third party, which may contribute to bias. For example, Schneider et al. used a very simple pain measurement instrument, the 7-point-Likert-Scale, with anchors: no pain, very low, low, moderate, strong, very strong, and unbearable (24). However, the use of uncommon evaluation tools makes it difficult to compare results between studies.

Notably, in all analyzed studies, pain decreased over time in treatment and control groups (18, 20, 21). Complaints in degenerative diseases of the spine generally have a chronically relapsing course with periods of exacerbation, then improvement. The latter can be accelerated with the help of medication, physiotherapy, manual therapy, and exercises. Similarly, focused ESWT significantly reduced pain and improved functional impairment immediately after the procedures and 1 month later. At the 3-month follow-up, the results remained better in the experimental compared to the control groups, despite minimal pain levels in both. None of the studies followed the treated patients long-term, and this is precisely where the focused shockwave has the potential for a significantly better outcome.

Patients who are not indicated for surgery but are still unresponsive to conservative treatment may benefit from focused ESWT to relieve pain. As an alternative to corticosteroid infiltrations, this approach dismisses the possibility of complications such as infection, hematoma, vessel injury, intravascular drug administration, hypertension, glucose intolerance, and osteoporosis development (25). The focused ESWT could also be combined with medical therapy and exercises (20). Despite the differences between these few studies, the findings show a significant reduction in low back pain and disability. However, none have a high level of evidence, treatment protocols are still not established, and sample sizes are small.

Several systematic reviews with meta-analyses of randomized controlled trials for ESWT of low back pain have been published (Table 6). None of them reliably confirm the effectiveness of the therapeutic approach despite the good results evident in each clinical trial. It is important to note that these reviews do not analyze separately or compare the radial against focused modality. Contrary to the results with radial ESWT, the focused devices are more promising in the context of the precise targeting and dosing of energy deep within the human body to the pathological process. However, only a few studies with a small number of patients and varying treatment protocols exist to make an unambiguous conclusion about the effectiveness of the therapy and the risk of complications. All future trials necessitate approving objective methods for assessment and establishing uniform treatment parameters.

**Table 6:** Systematic review with meta-analyses of randomized controlled trials of ESWT.

Author	Year	Study	Limitations
Yue et al. (40)	2021	Systematic review and meta-analysis of RCTs	8 radial ESWT/1 focused ESWT/1 vibrotherapy
Li et al. (41)	2022	Systematic review and meta-analysis of RCTs	11 radial ESWT/2 focused ESWT/1 vibrotherapy
Ma et al. (42)	2022	Systematic review and meta-analysis of RCTs	12 radial ESWT/1 focused ESWT/1 vibrotherapy
Wu et al. (43)	2023	Systematic review and meta-analysis of non-RCT and RCTs	18 radial ESWT/3 focused ESWT/1 vibrotherapy
Liu et al. (44)	2023	Systematic review and meta-analysis of RCTs	9 radial ESWT/2 focused ESWT/1 vibrotherapy

## Conclusion

Focused ESWT is a modern physiotherapeutic method that can potentially treat a broad spectrum of conditions responsible for low back pain. Despite the small number of low-evidence studies, there is sufficient data on the effectiveness and safety of this therapeutic modality. With future well-designed trials, the bias risks would be diminished, the indications for its use would expand, and the treatment protocols would be clarified.

## Data Availability

The original contributions presented in the study are included in the article/supplementary material, further inquiries can be directed to the corresponding author.
